# Effects of Low or High Dosages of Dietary Sodium Butyrate on the Growth and Health of the Liver and Intestine of Largemouth Bass, *Micropterus salmoides*

**DOI:** 10.1155/2022/6173245

**Published:** 2022-10-22

**Authors:** Yiyang Ge, Shibin Yao, Ye Shi, Chunfang Cai, Chengrui Wang, Ping Wu, Xiamin Cao, Yuantu Ye

**Affiliations:** School of Biology & Basic Medical Science, Soochow University, Suzhou 215123, China

## Abstract

The concentration of butyric acid in the intestine increased with the increase in the content of fermentable dietary fibre; however, the potential physiological impact of a high dose of butyric acid on fish has not been sufficiently studied. The aim of this study was to investigate the effect of two dosages of butyric acid on the growth and health of the liver and intestine of the largemouth bass (*Micropterus salmoides)*. Sodium butyrate (SB) was added to the diet at 0 g/kg (CON), 2 g/kg (SB2), and 20 g/kg (SB20), and the juvenile largemouth bass were fed to apparent satiation for 56 days. No significant difference was observed in the specific growth rate or hepatosomatic index among the groups (*P* > 0.05). The concentration of *β*-hydroxybutyric acid in the liver, the activities of alanine aminotransferase, aspartate aminotransferase, and alkaline phosphatase, and the concentrations of triglyceride and total cholesterol in serum increased significantly in the SB20 group compared to the CON group (*P* < 0.05). The relative expression of *fas*, *acc*, *il1b*, *nfkb*, and *tnfa* in the liver of the SB20 groups was also significantly higher than that of the CON group (*P* < 0.05). The above indicators in the group SB2 had similar change tendencies. The expression of *nfkb* and *il1b* in the intestine of both the SB2 and SB20 groups was significantly downregulated compared with that in the CON group (*P* < 0.05). The size of hepatocytes was enlarged, and the intracellular lipid droplets and the degree of hepatic fibrosis were increased in the SB20 group compared to the CON group. There was no significant difference in intestinal morphology among the groups. The above results indicated that neither 2 g/kg nor 20 g/kg SB had a positive effect on the growth of largemouth bass, while a high dosage of SB induced liver fat accumulation and fibrosis.

## 1. Introduction

After the positive effect of dietary sodium butyrate (SB) on terrestrial animals had been widely reported, studies on aquatic animals have also shown that SB improved growth performance [1–6], liver antioxidant capacity [5], intestinal structure integrity [2, 4–7], and decreased fat accumulation [8] . However, the positive effect of SB is not static [9] or may be dose-dependent [1, 10]. A study in a Caco-2 cell monolayer model showed that excessive butyrate may induce severe intestinal epithelial cell apoptosis and disrupt the intestinal barrier [11]. In mouse model, SB was confirmed to contribute to liver steatosis and fibrosis [12].

It was found that fatty liver [13, 14] and intestinal tissue damage [15] could be caused when fishmeal in the diet was replaced by plant protein sources. Dietary fibre (DF), which is rich in plant ingredients, has been suggested as a potential pathogenic agent in recent years [12, 16]. However, the pathogenic mechanism remains unclear. Butyric acid could be produced by fermentation of DF in the intestine [17, 18], and the yield of butyric acid was prominent when fermentable DF was high in the diet [19]. Therefore, it can be deduced that excessive butyric acid may contribute to liver and intestinal tissue damage in fish.

Farmed fish, including largemouth bass (*Micropterus salmoides)*, suffer from fatty liver disease and enteritis. With largemouth bass as a model animal, the aim of this study was to investigate the effects of different levels of SB on the health of the liver and intestine as well as the growth performance of fish. The results are helpful for better understanding the physiological effects of butyric acid and may also contribute to revealing the antinutritional mechanism of DF, ultimately benefiting fish farming.

## 2. Material and Methods

### 2.1. Ethical Statement

Procedures in this study were carried out in accordance with the “Guiding Principles in the Care and Use of Animals (China)” and standard operating procedures of Provincial Aquatic Animal Nutrition Key Laboratory of Soochow University. The ethical treatment of animals used in this study was approved by the Animal Welfare Ethics Committee of Soochow University (Approval No. SUDA20220810A01).

### 2.2. Diet

Casein and dextrin were purchased from Henan Gaobao Industrial Co., Ltd. (Zhengzhou, China), and the other diet ingredients were provided by Guangdong Yuehai Feed Co., Ltd. (Zhanjiang, China). According to the daily intake dosage of SB of experimental animals in the study by Lupton [12], the high dosage of SB (guaranteed reagent, purity >98%) in the diet was set at 20 g/kg (SB20 group). The low dosage was set at 2 g/kg (SB2 group) according to Liu et al. [5] and Dawood et al. [20]. All feed ingredients were ground to pass through a 60 mesh sieve, weighed according to the formulation, and mixed thoroughly. Then, 250 mL/kg distilled water was added and mixed again. The pellets were 2 mm in diameter and 5 mm in length and were made with an assembled machine. After air drying to a moisture content of less than 100 g/kg, the pellets were stored at -20°C. The diet formulation and proximate composition are shown in Table 1.

### 2.3. Feeding and Fish Management

The feeding trial was performed at the Graduate Workstation of Suzhou Yangchenghu National Modern Agricultural Demonstration Zone Development Co., Ltd. Juvenile largemouth bass were purchased from Jinchengfu Fishery Technology Co., Ltd. (Suzhou, China). Fish were reared in indoor cement ponds for 3 weeks and were fed the CON diet during this period. A similar size of 135 fish (average body weight 12.3 g/fish) was selected and randomly divided into 9 polyethylene tanks containing 300 L water, with 15 fish in each tank. The fish were fed the CON diet for another week and weighed again in a fasting state, and the total weight of each tank was adjusted to make the coefficient of variation <3% among tanks. Then, each experimental diet was randomly fed to three tanks of fish to apparent satiety at 7 : 30 and 16 : 30. Feed intake was calculated weekly. One-third of the water in the lower layer of the tank as well as the faeces was drawn by a siphon at 9 : 00 every day, and then fresh water was immediately input. A natural photoperiod with a light intensity above the water surface of approximately 800 lux was used at noon. The pH of the water was 7.5-7.8, the dissolved oxygen was >6.5 mg/L, and the ammonia nitrogen was <0.1 mg/L. The feeding trial lasted for 56 days.

### 2.4. Sampling and Sample Preparation

After 56 days of feeding, sampling was performed in a state of fasting. The fish were quickly netted, anaesthetized with 200 mg/L eugenol, and individually weighed, and the body length was measured. Three fish from each tank were randomly taken and dissected on ice under sterile conditions. The liver and hindgut tissues were sampled, washed with 0.01 mmol/L PBS (pH 7.2), placed in RNase-free centrifuge tubes, frozen in liquid nitrogen, and stored at -80°C for gene expression analysis. Three more fish were dissected and weighed for liver weight, and then the whole fish were homogenized and stored at -50°C for body composition analysis. The remaining 9 fish were utilized to draw blood. The blood was placed in a 1.5 mL centrifuge tube, stood at 4°C for 6 h, and centrifuged at 4000 rpm at 4°C for 10 min, and the serum was collected and stored at -80°C for subsequent serum biochemical analysis. These 9 fish were dissected on ice, and the liver was collected and weighed. After that, the liver and hindgut of three fish from each tank were taken, washed with 0.01 mmol/L PBS (pH 7.2), absorbed surface moisture with facial tissue, and fixed in 4% formaldehyde solution for histomorphological analysis; the liver and hindgut tissues of the other 6 fish from each tank were placed in a 10 mL tube, frozen with liquid nitrogen, and stored at -80°C for tissue biochemical analysis.

### 2.5. Analysis Method

#### 2.5.1. Diet and Fish Proximate Composition

Samples of whole fish were ground and freeze-dried (LGJ-18, Sihuangqihang Technology Co., Ltd., Beijing, China) to calculate moisture content. Moisture in the diet was measured by drying at 105°C to constant weight (DHG-9055A, Shanghai Yiheng Scientific Instrument Co., Ltd., China). The contents of crude protein, lipid, and ash in the dry matter were measured by the Kjeldahl method (GB/T 6432-2018, Kjeldahl nitrogen determinator: SKD-1000, Shanghai Peiou Analytical Instruments Co., Ltd., China; Digester: LNK-872, Jiangsu Yixing Science and Education Instrument Research Institute, China), the Soxhlet method (GB/T 6433-2006, glass Soxhlet extractor; thermostatic water bath equipment: DK-S26, Shanghai Jinghong Experimental Equipment Co., Ltd., China), and the burning method (GB/T 6438-2007, Muffle furnace: 8-10TP, Shanghai Huitai Instrument Manufacturing Co., Ltd., China), respectively.

#### 2.5.2. Serum Biochemistry and Liver Composition

An Abbott automatic blood biochemical analyser (Abbott c8000, USA) was used to determine the serum aspartate aminotransferase (AST), glutamate pyruvate aminotransferase (ALT), alkaline phosphatase (ALP), total triglyceride (TG), total cholesterol (TC), high density lipoprotein cholesterol (HDL-C), and low density lipoprotein cholesterol (HDL-C) according to the instructions of the kit, which was purchased from Meikang Biotechnology Co., Ltd. (Ningbo, China). The content of *β*-hydroxybutyric acid (*β*-HB) in the liver was extracted according to the method reported by Gao and Wu [21] and determined by a kit produced by Suzhou Grete Biomedical Co., Ltd. (Suzhou, China). The protein content of the liver was determined using a kit produced by Beyotime Biotechnology Co., Ltd. (Shanghai, China). The lipids in the liver were extracted and determined in accordance with the method reported by Staessen et al. [22]. The collagen fibre content in the liver was determined with Fish COL ELISA Kit (Sanjia, China).

#### 2.5.3. Related Expression of Lipid Metabolism and Inflammatory Genes

An appropriate amount of liver and hindgut tissue was thoroughly ground in the presence of liquid nitrogen. Total RNA was extracted following the instructions of the EASYspin plus kit (Beijing Adlai Biotechnology Co., Ltd., China). First-strand cDNA was synthesized using a 5X All-In-One RT MasterMix Kit (with AccuRT Genomic DNA Removal Kit, abmGood) and stored at -20°C. The mRNA expression of *fas*, *cpt1*, *acc*, *nfkb*, *il1b*, and *tnfa*, whose primer sequences are shown in Table 2, was determined by a relative quantitative method following the instructions of Taq Pro Universal SYBR qPCR Master Mix (Vazyme, Nanjing, China). *β*-Actin was used as an internal reference gene.

#### 2.5.4. Histological Morphology Analysis

The liver and hindgut tissues were removed from the formaldehyde solution, and slices were made following routine histological procedures and stained with haematoxylin eosin (H&E). The liver tissue sections were also stained with Masson and oil red O. The histological sections were observed with an optical microscope (Olympus BX51), and pictures were taken with an image acquisition system (SmartV550D, Jiangsu JieDa Technology Development Co., Ltd., China).

### 2.6. Calculation and Statistical Analysis

Weight gain rate (WGR), specific growth rate (SGR), feed conversion rate (FCR), feed intake (FI), feed intake rate (FIR), condition factor (CF), and hepatopancreas somatic indices (HSI) were calculated as follows:

(1) $$\begin{array}{c} \text{Weight gain rate }\left(\text{WGR},\%\right)=\frac{\text{Wf}-\text{Wi}}{\text{Wi}}\times 100,\\ \text{Specific growth rate }\left(\text{SGR},\frac{\%}{\text{d}}\right)=\frac{\left[\ln\left(\text{Wf}\right)-\ln\left(\text{Wi}\right)\right]}{\text{days}}\times 100,\\ \text{Feed conversion rate }\left(\text{FCR}\right)=\frac{\text{Wd}}{\text{Wf}-\text{Wi}},\\ \text{FI }\left(\frac{\text{g}}{\text{fish}}\right)=\frac{\text{Wd}}{\text{No}.\text{of fish}},\\ \text{Feed intake rate }\left(\text{FIR},\frac{\%}{\text{d}}\right)=\frac{\text{Wd}}{\left[\text{days}\times \left(\text{Wf}+\text{Wi}\right)\right./2}\times 100,\\ \text{Condition factor }\left(\text{CF},\%\right)=\frac{\text{BW}}{\left(\text{BL}\right)3}\times 100,\\ \text{Hepatosomatic index }\left(\text{HSI},\%\right)=\frac{\text{LW}}{\text{BW}}\times 100, \end{array}$$

where Wf (g/fish) is the mean final body weight, Wi (g/fish) is the mean initial body weight, Wd (g) is the weight of the intake diet in each tank, BW (g) is the individual body weight, BL (cm) is the fish body length, and LW is the liver weight (g).

All results are expressed as the mean ± standard deviations. One-way analysis of variance (ANOVA) was used to examine the difference among groups. Tukey's multiple comparison test was used when the variance was homogeneous, and Tamhane's T2 test was used when the variance was nonhomogeneous. Statistical analysis was performed by IBM SPSS 23, and the significance level was set at *P* < 0.05.

## 3. Results

### 3.1. Growth Performance and Body Condition

As shown in Table 3, there was no significant difference in FIR, WG, SGR, or FCR among the groups (*P* > 0.05). The FI of the SB20 group was significantly lower than that of the CON group and the SB2 group (*P* < 0.05).

Supplementation with SB showed no significant effects on the body content of moisture, crude protein, lipid, and ash (*P* > 0.05) regardless of the dosage (Table 4). The CF decreased while the HSI increased with increasing SB dosage, but not significantly (*P* > 0.05).

### 3.2. Biochemical Indices of Serum and Liver

**Figure 1:**
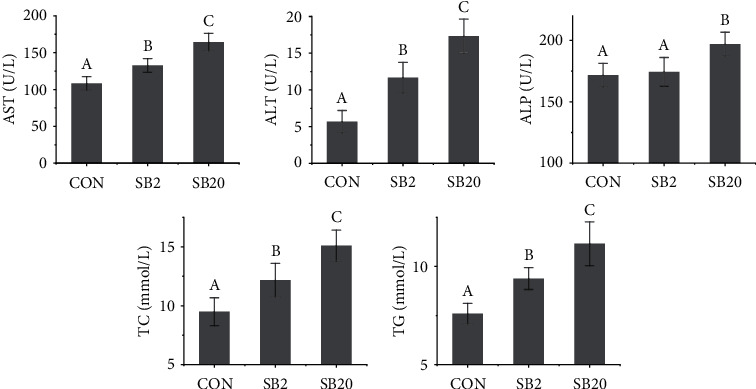
Four effects of two dosages of sodium butyrate on serum biochemistry of largemouth bass, *Micropterus salmoides.* AST: aspartate aminotransferase; ALT: alanine aminotransferase; ALP: alkaline phosphatase; TC: total cholesterol; TG: total triglycerides. Different letters above the bars represent significant differences (*P* < 0.05, *n* = 3).

The activities of AST and ALT in the serum increased substantially with increasing SB dosage (*P* < 0.05, Figure 1). The activity of ALP in the serum of the SB20 group was considerably higher than that of the SB2 group and the CON group (*P* < 0.05). No significant difference was observed between the SB2 group and the CON group (*P* > 0.05). The serum TG concentration was 22% and 43% higher than that of the SB2 group and the CON group, respectively, while the TC concentration of the SB20 group was 26% and 65% higher, respectively (*P* < 0.05).

**Figure 2:**
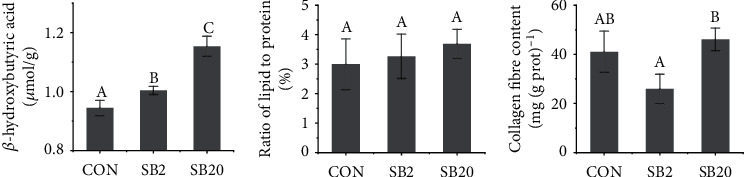
Effects of two dosages of sodium butyrate on liver biochemical indices of largemouth bass, *Micropterus salmoides.* Different letters above the bars represent significant differences (*P* < 0.05, *n* = 3).

With the increase in SB in the diet, the concentration of *β*-HB in the liver increased significantly (*P* < 0.05, Figure 2). The ratio of lipid to protein in the liver showed a trend of increasing, but not significantly (*P* > 0.05). The collagen fibre content in the SB20 group was higher, while in the SB2 group lower than that in the CON group, but not significantly (*P* > 0.05, Figure 2).

### 3.3. mRNA Expression of Genes Related to Lipid Metabolism and Inflammation

**Figure 3:**
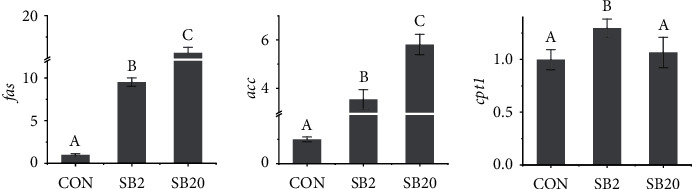
Effects of two dosages of sodium butyrate on the relative expression of liver lipid synthesis-related genes in largemouth bass, *Micropterus salmoides. fas*: encode fatty acid synthase; *acc*: encode acetyl-CoA carboxylase; *cpt1*: encode carnitine palmityl transferase 1. Different letters above the bars represent significant differences (*P* < 0.05, *n* = 3).

As shown in Figure 3, with the increase in the dosage of SB, the relative expression of *fas* and *acc* mRNA in the liver increased significantly. The expression of *cpt1* in the SB2 group was higher than that in the CON group and the SB20 group (*P* < 0.05), and there was no significant difference between the latter two groups (*P* > 0.05).

**Figure 4:**
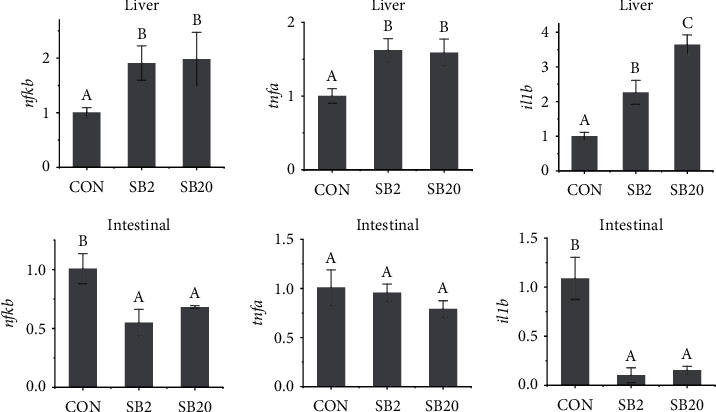
Effects of two dosages of sodium butyrate on the relative expression of liver and intestine inflammation-related genes in largemouth bass, *Micropterus salmoides*. *nfkb*: encode nuclear factor-*κβ*; *tnfa*: encode tumour necrosis factor-*α*; *il1b*: encode interleukin-1*β*. Different letters above the bars represent significant differences (*P* < 0.05, *n* = 3).

The mRNA expression of *nfkb*, *tnfa*, and *il1b* in the liver of fish fed a diet containing SB was significantly higher than that of the CON group, and the expression of *il-1b* increased with increasing SB dosage (*P* < 0.05, Figure 4). In intestinal tissue, however, the expression levels of *nfkb* and *il1b* in fish fed with a diet containing SB were significantly lower than those in the CON group (*P* < 0.05), and there was no significant difference between the SB20 group and the SB2 group (*P* > 0.05). No significant difference in *tnfa* expression in intestinal tissue was observed among the groups (*P* > 0.05).

### 3.4. Histological Analysis

**Figure 5:**
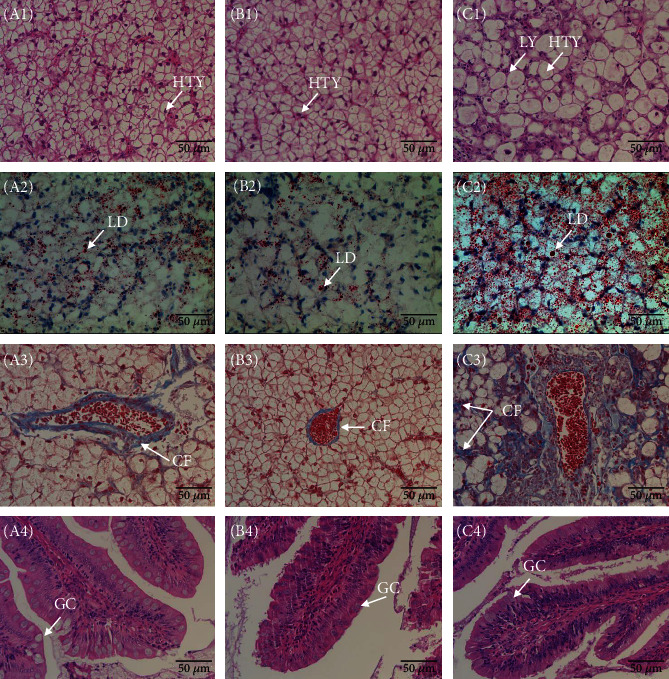
Photo of liver (rows 1 to 3) and intestinal sections (row 4) of largemouth bass, *Micropterus salmoides*, fed the CON (A1-A4), SB2 (B1-B4), and SB20 (C1-C4) diets. HTY: hepatocytes; LY: lymphokines; LD: lipid droplets (red); CF: collagen fibre (blue); GC: goblet cells.

Compared to the CON group (Figure 5), the liver sections of the SB20 group showed enlargement of hepatocytes, increased amount of intracellular lipid droplets, blurred cell edges, and lymphocyte infiltration (Figure 5, C1 and C2). The liver cells in the SB2 group showed clear edges and nuclei and no obvious histopathological changes or fat accumulation (Figure 5, B1 and B2). Masson-stained liver tissue sections showed strong blue signals, which showed collagen fibre, appearing only near the portal area but not among hepatocytes in the CON and SB2 groups. In the SB20 group, the blue signals were obvious among hepatocytes (Figure 5 C3). No obvious difference was observed in the integrity of intestinal tissue, the height and width of fold, infiltration of lymphocytes, number of goblet cell, etc. among the groups (data not given).

## 4. Discussion

Studies have shown that fish growth performance could be improved by supplementation of butyrate to the diet; however, the appropriate amount seems to vary among fish species. Jesus et al. [3] reported that the growth performance of Nile tilapia (*Oreochromis niloticus*) was improved when 5 g/kg SB was added to the diet. Omosowone et al. [23] suggested that 2 g/kg butyric acid in *Clarias gariepinus* and 1.5 g/kg butyric acid in *Oreochromis niloticus* were optimal for growth. Liu et al. [5] reported that appropriate dietary supplementation of SB was 2 g/kg to juvenile grass carp (*Ctenopharyngodon idella*) for growth, while Tian et al. [6] recommended 0.1608 g/kg SB (microencapsulated) for the growth of young grass carp. Studies also showed that the growth rate decreased significantly when the dosage of butyrate was beyond the optimal value [1, 4], and no improvement in growth was observed in grass carp (*Ctenopharyngodon idella*) fed with a diet containing 0.5 g/kg SB [8] or in juvenile giant grouper, *Epinephelus lanceolatus*, fed with a diet containing 10 g kg^−1^ butyric acid [9]. In this study, no significant changes in growth performance occurred in either the SB2 or SB20 groups, in which 2 g/kg and 20 g/kg SB was supplied, respectively. The current results, together with the above reports, suggested that the growth-promoting effect of butyric acid might occur only under specific conditions.

Few studies have evaluated the effect of SB as high as 20 g/kg on the growth and physiology of animals, and limited results have shown that the addition of SB up to 50 g/kg has no negative effect on body weights and food intake in mice [24]. No statistically significant change in SGR was noticed in the SB20 group in this study. However, the FI of the SB20 group decreased significantly compared with that of the CON group and the SB2 group. Butyric acid stimulates the secretion of peptide YY (PYY) [25], and PYY acts on the peripheral and central nervous systems through the brain gut axis and contributes to a feeling of satiety [26, 27], which might be the reason for the decline in FI in the SB20 group. The decreased FI further led to decreasing SGR, although not significantly.

Many studies have shown that oral administration of short-chain fatty acids (including butyric acid) can attenuate fat deposition by reducing lipogenesis and enhancing lipolysis in different tissues [8, 28, 29]. However, the effect of butyric acid on lipid metabolism may be completely opposite depending on the dose. Zhao et al. [30] noticed that in chicken adipocytes, the fat droplets laden were enlarged accompanied by activation of lipogenic gene expression when butyrate was at a higher concentration (1 mM); however, the opposite response was observed at a lower concentration (0.01 mM). Zhang et al. [31] observed in pigs that intravenous SB increased fatty acid synthesis, decreased lipolysis in muscle tissue, and increased lipolysis in adipose tissue. In this study, histological sections showed obviously enhanced lipid deposition in the liver of the SB20 group compared to the CON group. The fat protein ratio, which was used to evaluate liver lipid degeneration [32], also showed an increasing trend in the SB20 group. Liver steatosis is usually accompanied by increases in TC and TG in serum [33, 34]. In this study, the contents of serum TG and TC increased in the SB20 group. All of the above results suggested the induction effect of high-dose SB on fatty liver, which to our knowledge has never been reported in fish. In mice, the direct induction of fatty liver with a high dosage of butyric acid has been reported [12]. *fas* and *acc* are key genes for de novo fatty acid synthesis. With the increase in SB in the diet, the mRNA expression of *fas* and *acc* in liver tissue was significantly upregulated. These results suggested that liver fat accumulation induced by high-dose SB might occur via de novo hypersynthesis of fatty acids, which has been observed in bovine mammary epithelial cells [35], dairy cows [36], and growing pigs [31].

Generally, butyric acid in the intestine enters the peripheral circulation through the hepatic portal vein and is finally oxidized in the liver to supply energy following the *β*-oxidation pathway [37, 38]. The current results showed that with increasing SB in the diet, the concentration of *β*-HB increased significantly, suggesting that butyrate was not completely oxidized under the experimental dosage and that ketone bodies accumulated in the liver. The strong ketogenic capacity of butyrate was also observed in humans [39]. Ketosis is closely associated with increased biomarkers of inflammation [40, 41], and inflammatory reactions induce not only fat accumulation [42, 43] but also fibrosis [44]. In this study, liver fibrosis was also noted in the SB20 group, while the mRNA expression of the inflammatory factor genes *nfkb*, *il1b*, and *tnfa* increased. These results indicated that the pathological changes in liver tissue might be attributed to the chronic inflammatory reaction induced by accumulated *β*-HB. Similar liver histopathological changes and inflammatory reactions were also observed in mice taking butyrate orally [12].

Hepatic steatosis and fibrosis are often accompanied by an increase in the enzyme activities of AST, ALT, and ALP in serum [45, 46]. In this study, the activity of AST, ALT, and ALP in the SB20 group was increased, which was consistent with the histopathological changes. Unexpectedly, the activity of AST and ALT together with the expression of *nfkb*, *il1b*, and *tnfa* in the SB2 group were also significantly higher than those in the CON group, indicating that liver tissue was also damaged to a certain extent, although the pathological changes in liver tissue were not obvious.

Studies have suggested that an appropriate amount of butyric acid is beneficial in resistance to intestinal tissue damage [2, 4–6] and downregulates the mRNA expression of proinflammatory factor genes [5], and excessive butyrate destroys mucosal barrier function [10]. In this study, there was no obvious abnormality in the intestinal tissue in either the SB2 group or the SB20 group. In addition, the mRNA expression of *nfkb* and *il1b* in the intestine was lower than that in the CON group. These results indicated that SB has a certain anti-inflammatory effect on the intestine, even at doses as high as 20 g/kg. Butyric acid can be produced from the fermentation of DF by bacteria in the intestine [18]. Therefore, intestinal epithelial cells may have a high tolerance to high concentrations of butyric acid, which may be the reason for the lack of obvious pathological changes in the intestine in the SB20 group.

In our previous study, it was noticed that high DF led to hepatic steatosis and fibrosis and enteritis in yellow catfish [16, 47]. DF can be fermented by microflora in the intestine and produce butyric acid [18]. The current results showed that a high dosage of SB caused liver fat accumulation accompanied by fibrosis. It can, therefore, be inferred that DF induced liver fat accumulation and tissue damage partly through the fermentation product butyric acid.

## 5. Conclusion

The addition of 2 g/kg and 20 g/kg SB to the diet did not improve the growth performance of largemouth bass, while 20 g/kg SB caused liver lipid accumulation and fibrosis, which might be attributed to de novo hypersynthesis of fatty acids and the inflammatory reaction.

## Figures and Tables

**Table 1:** Formulation and proximate composition of the experimental diets (g/kg, air dry basis).

	CON	SB2	SB20
Ingredients			
Fish meal	350	350	350
Casein	280	280	280
Dextrin	200	200	200
Squid paste	20	20	20
Fish oil	20	20	20
Soybean oil	30	30	30
Soy lecithin	20	20	20
Vitamin and mineral premix	25	25	25
Calcium dihydrogen phosphate	15	15	15
Sodium butyrate	0	2	20
Zeolite powder	40	38	20
Proximate composition			
Moisture	8.3	9.0	8.8
Crude lipid	9.4	8.8	9.4
Crude protein	46.9	47.4	46.3
Ash	11.1	10.7	11.1

Vitamin and mineral premix for largemouth mouth bass were provided by Beijing Sangpu Biochemical Technology Co., Ltd. (in China).

**Table 2:** Primer sequences for quantitative real-time PCR.

Primer sequence	(5′-3′) Fordward primer	(5′-3′) reverse primer
*β-actin*	AAAGGGAAATCGTGCGTGAC	AAGGAAGGCTGGAAGAGGG
*fas*	CAGCCCTTGACTCATTCCG	CGCAGACTACGACCCGACAG
*cpt1*	TTCCCCTTTATTGACTTTGGC	AGAACTTCCCTTTGTCCCTGTAA
*acc*	ATCCCTCTTTGCCACTGTTG	GAGGTGATGTTGCTCGCATA
*tnfa*	CTAGTGAAGAACCAGATTGT	AGGAGACTCTGAACGATG
*il1b*	TTGCCATAGAGAGGTTTA	ACACTATATGCTCTTCCA
*nfkb*	GCTGCTCGTTGTCGTGAATA	CCACTTCACCCCTGATTGACT

*Note: β-actin* encodes *β*-actin; *fas* encodes fatty acid synthase; *cpt1* encodes carnitine palmitoyltransferase 1; *acc* encodes acetyl-CoA carboxylase; *tnfa* encodes tumour necrosis factor *α*, *il1b* encodes interleukin-1*β*; *nfkb* encodes nuclear factor kappa-*β*.

**Table 3:** Effects of two dosages of sodium butyrate on the growth performance of largemouth bass, *Micropterus salmoides.*

Item	CON	SB2	SB20
Initial body weight (g/fish)	16.22 ± 0.07^a^	16.33 ± 0.16^a^	16.26 ± 0.10^a^
Finial body weight (g/fish)	67.44 ± 1.84^a^	66.50 ± 4.59^a^	62.56 ± 5.05^a^
Weight gain rate (%)	315.73 ± 13.00^a^	307.33 ± 32.22^a^	284.47 ± 28.68^a^
Specific growth rate (%/d)	2.54 ± 0.06^a^	2.50 ± 0.14^a^	2.40 ± 0.13^a^
Feed intake (g/fish)	42.34 ± 1.26^b^	42.62 ± 1.54^b^	37.59 ± 2.06^a^
Feed intake rate (%/d)	1.80 ± 0.07^a^	1.84 ± 0.09^a^	1.71 ± 0.18^a^
Feed conversion rate	0.82 ± 0.04^a^	0.85 ± 0.07^a^	0.83 ± 0.12^a^
Condition factor (%)	2.42 ± 0.08^a^	2.40 ± 0.15^a^	2.29 ± 0.14^a^
Hepatosomatic index (%)	0.042 ± 0.006^a^	0.044 ± 0.004^a^	0.046 ± 0.005^a^

Note: different superscripts in the same row of data indicate significant differences (*P* < 0.05, *n* = 3).

**Table 4:** Effects of two dosages of sodium butyrate on the body composition of largemouth bass, *Micropterus salmoides.*

Item	CON	SB2	SB20
Moisture (%)	68.65 ± 0.78^a^	68.79 ± 0.55^a^	69.01 ± 0.62^a^
Crude ash (dry matter, %)	14.79 ± 1.13^a^	13.89 ± 1.85^a^	13.72 ± 0.59^a^
Crude fat (dry matter, %)	23.64 ± 1.54^a^	23.88 ± 0.99^a^	23.48 ± 0.90^a^
Crude protein (dry matter, %)	58.80 ± 0.60^a^	57.26 ± 1.63^a^	57.41 ± 0.75^a^

Note: different superscripts in the same row of data indicate significant differences (*P* < 0.05, *n* = 3).

## Data Availability

The data that support the findings of this study are available from the first author, Yiyang Ge (438748144@qq.com).
